# SOURCE: A Registry-Based Prediction Model for Overall Survival in Patients with Metastatic Oesophageal or Gastric Cancer

**DOI:** 10.3390/cancers11020187

**Published:** 2019-02-05

**Authors:** Héctor G. van den Boorn, Ameen Abu-Hanna, Emil ter Veer, Jessy Joy van Kleef, Florian Lordick, Michael Stahl, Jaffer A. Ajani, Rosine Guimbaud, Se Hoon Park, Susan J. Dutton, Yung-Jue Bang, Narikazu Boku, Nadia Haj Mohammad, Mirjam A. G. Sprangers, Rob H. A. Verhoeven, Aeilko H. Zwinderman, Martijn G. H. van Oijen, Hanneke W. M. van Laarhoven

**Affiliations:** 1 Department of Medical Oncology, Cancer Center Amsterdam, Amsterdam UMC, University of Amsterdam, 1105 AZ Amsterdam, The Netherlands; h.g.vandenboorn@amc.uva.nl (H.G.v.d.B.); e.terveer@amc.uva.nl (E.t.V.); j.j.vankleef@amc.uva.nl (J.J.v.K.); m.g.vanoijen@amc.uva.nl (M.G.H.v.O.); 2 Department of Medical Informatics, Amsterdam UMC, University of Amsterdam, 1105 AZ Amsterdam, The Netherlands; a.abu-hanna@amc.uva.nl; 3 1st Medical Department, University Cancer Center Leipzig (UCCL), University Hospital Leipzig, 04103 Leipzig, Germany; florian.lordick@medizin.uni-leipzig.de; 4 Department of Medical Oncology and Hematology, Kliniken Essen-Mitte, 45136 Essen, Germany; m.stahl@kliniken-essen-mitte.de; 5 Department of Gastrointestinal Medical Oncology, The University of Texas MD Anderson Cancer Center, Houston, TX 77030, USA; jajani@mdanderson.org; 6 Department of Medical Oncology, Centre Hospitalo-Univeristaire de Toulouse, 31400 Toulouse, France; guimbaud.r@chu-toulouse.fr; 7 University School of Medicine, Samsung Medical Center, Sungkyunkwan, Seoul 06351, Korea; hematoma@skku.edu; 8 Oxford Clinical Trials Research Unit and Centre for Statistics in Medicine, University of Oxford, Oxford OX1 2JD, UK; susan.dutton@csm.ox.ac.uk; 9 Seoul National University College of Medicine, Seoul National University Hospital, Seoul 03080, Korea; bangyj@snu.ac.kr; 10 Department of Gastrointestinal Medical Oncology Division, National Cancer Center Hospital, Tokyo 104-0045, Japan; nboku@ncc.go.jp; 11 Department of Medical Oncology, UMC Utrecht, Utrecht University, 3584 CX Utrecht, The Netherlands; n.hajmohammad@umcutrecht.nl; 12 Department of Medical Psychology, Amsterdam UMC, University of Amsterdam, 1105 AZ Amsterdam, The Netherlands; m.a.sprangers@amc.uva.nl; 13 Netherlands Comprehensive Cancer Organization (IKNL), 5612 HZ Eindhoven, The Netherlands; r.verhoeven@iknl.nl; 14 Department of Surgery, Radboud University Medical Centre, 6525 GA Nijmegen, The Netherlands; 15 Department of Clinical Epidemiology, Biostatistics and Bioinformatics, Amsterdam UMC, University of Amsterdam, 1105 AZ Amsterdam, The Netherlands; a.h.zwinderman@amc.uva.nl

**Keywords:** prediction model, oesophageal cancer, gastric cancer, metastasis, Cox regression, Delphi consensus

## Abstract

Prediction models are only sparsely available for metastatic oesophagogastric cancer. Because treatment in this setting is often preference-based, decision-making with the aid of a prediction model is wanted. The aim of this study is to construct a prediction model, called SOURCE, for the overall survival in patients with metastatic oesophagogastric cancer. Data from patients with metastatic oesophageal (*n* = 8010) or gastric (*n* = 4763) cancer diagnosed during 2005–2015 were retrieved from the nationwide Netherlands cancer registry. A multivariate Cox regression model was created to predict overall survival for various treatments. Predictor selection was performed via the Akaike Information Criterion and a Delphi consensus among experts in palliative oesophagogastric cancer. Validation was performed according to a temporal internal-external scheme. The predictive quality was assessed with the concordance-index (c-index) and calibration. The model c-indices showed consistent discriminative ability during validation: 0.71 for oesophageal cancer and 0.68 for gastric cancer. The calibration showed an average slope of 1.0 and intercept of 0.0 for both tumour locations, indicating a close agreement between predicted and observed survival. With a fair c-index and good calibration, SOURCE provides a solid foundation for further investigation in clinical practice to determine its added value in shared decision making.

## 1. Introduction

Patients with oesophageal or gastric cancer have a relatively poor prognosis. One of the main contributors to the low survival rates is the high prevalence of metastases [1]. Metastatic disease is reported to be present at diagnosis in around 20–30% of oesophageal and in 30–40% of gastric cancer patients [2,3]. Although treatments with curative intent are often not an option when a patient presents with metastatic disease, treatments such as systemic therapy may still prolong life and/or offer symptom relief [4]. Treatment guidelines show, however, that in certain cases best supportive care should be considered in patients with metastatic oesophagogastric cancer [5,6]. As treatment is not always associated with improvement of increased health-related quality of life, the best treatment choice for a particular patient may not be obvious [7].

Informing patients about their treatment options and the associated risks and benefits can therefore be difficult due to complexity of the patients’ disease and heterogeneity of outcomes [8]. Prediction models, however, can aid in this process and allow individualized decision making [9]. Over the years various prediction models have been developed to support this process, by predicting outcomes such as survival and recurrence in cancer patients. The Adjuvant! Online prediction model, for example, predicts survival in breast cancer patients on the basis of various demographic and clinical variables [10]. An important feature is the comparison of various treatments by displaying the added survival benefit. Recently a review of the prediction models for oesophageal and gastric cancer showing that nearly all prediction models available for oesophagogastric cancer are aimed at predicting survival after curative treatment [11]. Only two prediction models are available that are intended for patients with metastatic disease. The model by Jung et al. predicts overall survival based on a dataset of 239 South Korean patients with oesophageal squamous cell carcinoma. All patients were treated with either fluorouracil/cisplatin or capecitabine/cisplatin in a first-line setting [12]. The model was presented as a nomogram and predicts the one-year survival probability. For the model by Shiozaki et al., 64 patients with metastatic adenocarcinomas were included and all received chemotherapy followed by chemoradiation [13]. The model, intended for patients with favourable outcomes, was also presented as a nomogram and predicts the median overall survival time.

Given the restrictive inclusion criteria and small sample sizes, the generalisability of these models is likely to be limited which possibly hampers implementation in clinical practice. A model is needed that focuses on patients with metastatic disease and informs on the various treatment options which the patient is facing. 

It is therefore the aim of this study to create and evaluate a prediction model based on a large nationwide dataset for use in clinical practice, called SOURCE (Stimulating evidence based, personalized and tailored information provision to improve decision making after Oesophagogastric Cancer diagnosis). SOURCE is intended to predict overall survival for a variety of treatment options in a heterogeneous group of patients with metastatic oesophageal or gastric cancer.

## 2. Results

An overview of the metastatic oesophageal (*n* = 8010) and gastric cancer (*n* = 4763) patients whose data were used to create the prediction model, is given in Table 1. Additional characteristics of this cohort are available in Table S2.

### 2.1. Selected Predictors

Of the corresponding authors of 41 phase III trials who were invited, eight agreed to participate in the Delphi consensus and completed both rounds [14]. In round one, fourteen of the 56 predictors were retained and twenty-five were excluded. Additionally, seventeen predictors were selected by 20–50% of the experts, and eight new predictors were proposed by the experts. These 25 predictors were considered during the second consensus round. Finally, three predictors were included during the second consensus round. The total number of included predictors of the first and second round therefore is seventeen. The outcomes of the Delphi procedure are displayed in more detail in Table S1 and the final selection of the predictors determined by the consensus are displayed in Table 2 alongside the selected SOURCE predictors. The Delphi consensus procedure selected ten predictors which were unavailable in the NCR dataset and could therefore not be included in the list of pre-selected variables. Seven predictors selected in the Delphi consensus were available in the NCR, all of which were selected as predictors in the final SOURCE models.

### 2.2. Final Model Parameters

The model parameters of the resulting SOURCE model for overall survival in metastatic oesophageal cancer and metastatic gastric cancer are presented in Table 3; Table 4, respectively. 

**Figure 1:**
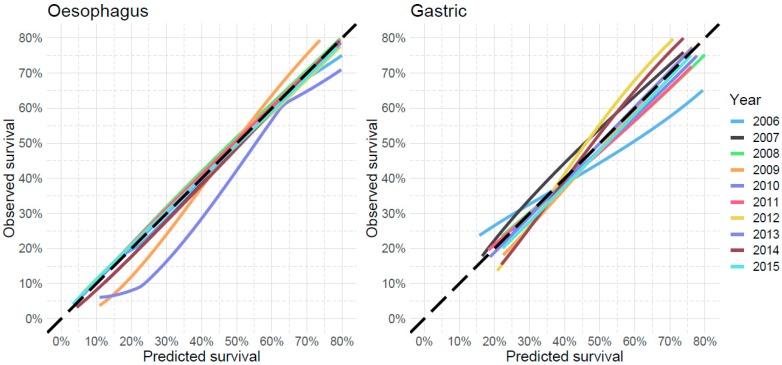
Calibration plots during temporal cross-validation for the oesophageal model (left) and the gastric model (right). The different lines indicate the correspondence between predicted and observed survival for various diagnosis years. The calibration plot was established at the median overall survival (5.1 months for oesophageal cancer and 3.9 months for gastric cancer). The dashed line indicates an ideal calibration line with an intercept of 0 and slope of 1.

**Figure 2:**
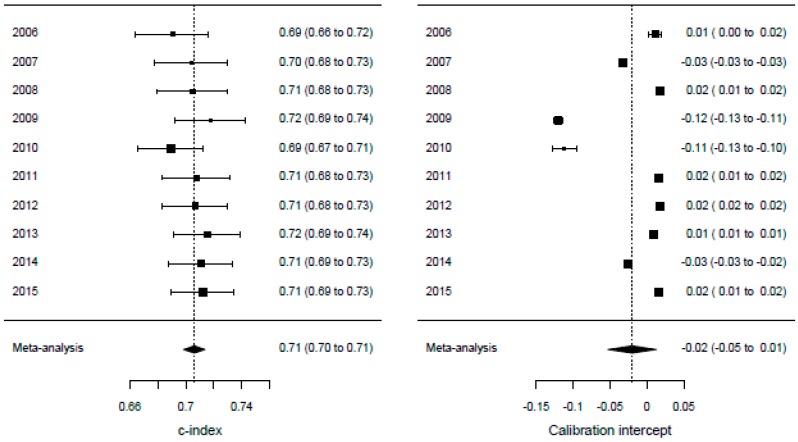

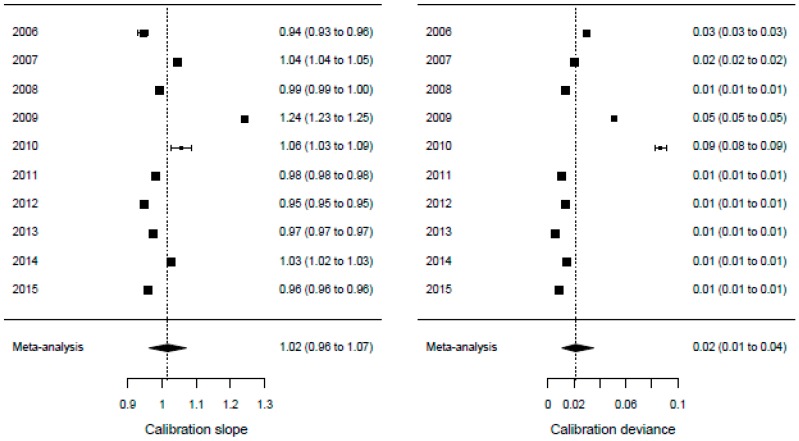
Meta-analysis for internal-external cross-validation (oesophagus). Each of the four panels shows the meta-analysis of the model outcomes for oesophageal cancer patients. The year indicates on which diagnosis year cohort the model is validated.

**Figure 3:**
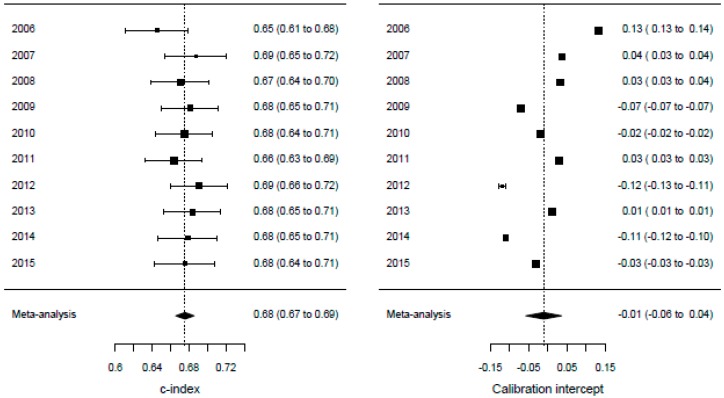

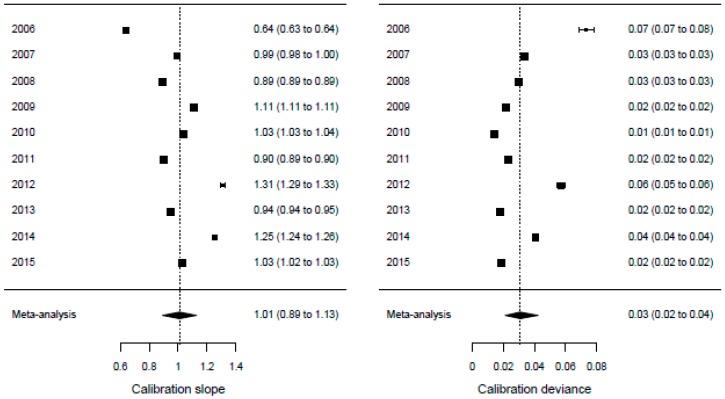
Meta-analysis for internal-external cross-validation (gastric). Each of the four panels shows the meta-analysis of the model outcomes for gastric cancer patients. The year indicates on which diagnosis year cohort the model is validated.

The performance measures for both the complete SOURCE model and the internal-external validation are shown in Table 5. The results show that the prediction model has a slightly better performance in oesophageal cancer than in gastric cancer. The calibration slopes and intercepts lie close to the optimal values of 1 and 0, respectively. While the performance measures are marginally lower during validation than in the complete model, the correspondence between both settings remain high. The calibration plots of the temporal validation cross-validations are shown in Figure 1. The meta-analyses of the model performance statistics are shown in Figure 2 for oesophageal cancer and in Figure 3 for gastric cancer. These figures show the performance statistics for each validation cohort.

## 3. Discussion

The SOURCE model presented in this paper is the first prediction model for survival outcome of metastatic oesophageal and gastric cancer patients that was created with a large (*n* = 12,773) nation-wide cohort, and includes treatment as a separate predictor. This allows for a flexible model enabling the provision of prognoses for various treatments and tumour locations within the upper gastrointestinal tract. Importantly, the predictors included in the SOURCE model are available in standard clinical care and do not require additional tests that may be cost prohibitive. The strengths of SOURCE lie in its clinical applicability, providing a model for all metastatic oesophagogastric cancer patients and including various treatment options. 

In creating SOURCE, various steps were undertaken to increase the quality of the model, its reproducibility and its robustness. First, the predictors were selected both by the bidirectional Akaike’s Information Criterion (AIC) procedure and a Delphi consensus procedure, thereby combining “the best of both worlds” including data-driven analysis and expert clinician-guided selection. Although not all proposed predictors were available in the current dataset, future models can be built using this selection. Secondly, a temporal internal-external cross-validation method was employed based on the year of diagnosis [15]. With this approach, advances in patient care and treatment are taken into account. In addition, this approach is comparable to a true external temporal validation where an existing prediction model is validated on new patients. Lastly, instead of a complete case analysis, which excludes patients with missing data and thereby could increase bias, we employed the robust multiple imputation method for handling missing data [16]. This not only has the advantage of dealing with uncertainty of the imputations, but can also be used for the transformation of specific variables, such as TNM staging, thus enabling a richer dataset on which the prediction model was based. With these methods, it is possible to obtain a more precise estimate of the model parameters while keeping the amount of over-fit small. 

Indeed, the SOURCE model showed a fairly discriminative ability, with a c-index of approximately 0.71 for oesophageal cancer and 0.68 for gastric cancer. Although certain other models were able to discriminate better between patients, it must be noted that our dataset was relatively homogenous, including patients with metastatic oesophagogastric cancer only [17,18]. Differentiating between survival outcomes of a rather homogeneous group of patients is more complex than differentiating between survival outcomes of patients with cancers from various primary origins and known large differences in survival. The model further shows an overall good average accordance between predicted and observed survival. These results remain consistent between the full model and the internal-external temporal cross-validation, thus indicating a lack of over-fit. Additional external validation with cohorts from other countries and more recent years is needed to further examine the robustness of the model. 

Some limitations of this study have to be acknowledged. First of all, ten predictors selected in the Delphi consensus were not represented in the dataset (see Table 2) and could therefore not be included in the final prediction model. Inclusion of at least some of these predictors, would likely have improved the model’s performance. One of the most important predictors of overall survival, performance status, was reported limitedly in the NCR only for the year 2015 and could therefore not be included in the analysis [19]. Further, the initial treatment variable lacks detail. Ideally, several therapies would be subdivided (such as various chemotherapy regimens) to enable a better fit of the model parameters. However, performance status and more detailed treatment information will be available more abundantly in future years and could become predictors in the prediction model. This stresses the need for intermittent updating of predictions models when new data becomes available to increase the model’s performance and keep up with the development of new treatment options over time. It can also be noted that the models display in some cases hazard ratio’s below 1 for cT and cN stages, implying an unexpected slight decrease of hazard compared to the cT1 and cN1 stages. We hypothesise that this may be caused by aggressive tumour behaviour resulting in a shorter overall survival in patients who developed metastases despite a low cT or cN stage. Lastly, SOURCE predicts overall survival at diagnosis. However, due to the nature of the registration process the dataset also erroneously included patients initially diagnosed as cM0 but whose staging was updated within six weeks to cM1 due to disease progression or the discovery of metastases during additional diagnostic testing. Consequently, patients who started treatment with curative intent, such as resections, may be overrepresented. Unfortunately, these patients could not be identified and excluded. Based on a detailed analysis of a subset of patients, we estimate this percentage to be small (~6%).

**Figure 4:**
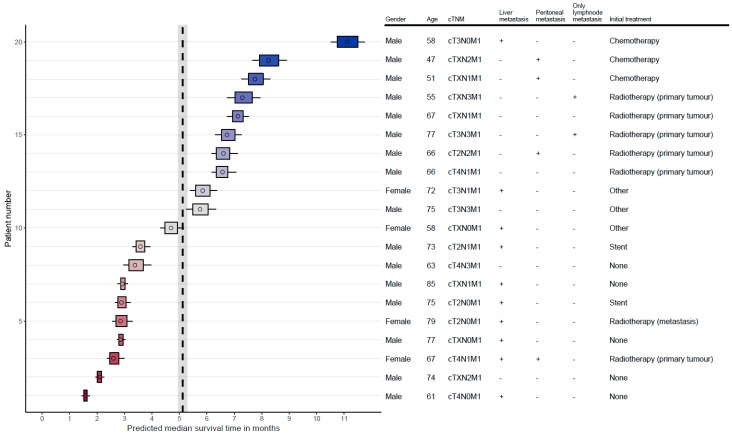
Predicted median survival times for metastatic oesophageal cancer. The figure demonstrates the practical applicability of the SOURCE model in individual patients. The diagram is based on a random sample of 20 patients in the dataset. The SOURCE model predicts median survival time with accompanying 50% confidence interval (bars) and 80% confidence intervals (lines). The dashed line indicates the observed median survival and confidence interval of all patients in the dataset. On the right, the patient characteristics are shown on which the predictions were based.

Use of the SOURCE model could be valuable and helpful in clinical practice and stimulate shared decision making. In shared decision making, well balanced provision of information is key [20]. The possibility to compare different treatment options, e.g., chemotherapy and best supportive care could stimulate shared decision making. Figure 4 shows how the SOURCE model can be applied to individual patients in practice, based on specific patient characteristics. The figure shows the model predictions as well as the uncertainty at patient level. In practice, it is possible to calculate multiple survival probabilities for a single patient by selecting various initial treatments. However, one must take care in selecting the therapies as not all treatment may be relevant for the patient. Additionally, the survival predictions may have an inherent selection bias that needs to be considered. Patients in the NCR dataset that received no treatment probably had a worse performance status than patients that did receive treatment. This may result in an underestimation for the prediction of survival for best supportive care. Although this effect is partly corrected by other predictors in the model, there may still be bias in the model predictions.

Thus, these statistics and other model outcomes could be used to inform patients and aid the decision process by showing the relative change in survival for individual patients between treatments. To allow for implementation in clinical practice, however, a visual format is needed. For this purpose, we have created an interactive web-interface for SOURCE [21]. Although a nomogram is commonly used to this end, this presentation format is unsuitable for SOURCE as it contains interaction variables. The web-interface also contains functionality to highlight viable treatment options on the basis of patient characteristics. This will aid the selection of relevant treatments for patients in SOURCE. After extensive testing in clinical practice, this SOURCE web-interface will be made freely available for the oncological community. 

## 4. Materials and Methods 

This report was written in accordance to the TRIPOD (Transparent Reporting of a multivariable prediction model for Individual Prognosis Or Diagnosis) guidelines [22]. Data of the prospectively maintained population-based Netherlands Cancer Registry (NCR) was used in the development and validation of the prediction model. The records of all 14,422 metastatic oesophageal and gastric cancer patients diagnosed between January 2005 and December 2015 were retrieved from the NCR. Patients with unknown follow-up (*n* = 4), patients who had T0 tumours (*n* = 5) and patients with cancer types other than carcinomas (*n* = 227) were excluded from further analysis. Additionally, patients who died within fourteen days after diagnosis (*n* = 697) were also excluded, because patients in such poor health would not likely use a prediction model. Patients with multiple primary tumours (*n* = 9) retained their initial tumour in the dataset, and subsequent tumours were excluded. Finally, patients whose only distant metastases were located in lymph nodes in the head or neck region, were excluded from further analyses (*n* = 707). These patients could be treated with a curative intent and therefore fall outside the scope of the prediction model. This left a total of 8010 oesophageal cancer patients and 4763 gastric cancer patients for inclusion in the dataset.

The outcome of the SOURCE prediction model is overall survival as it gives the most complete survival information for patients. It was measured from the date of diagnosis to the date of death, or the date of last follow up when the patient was censored.

The development of the SOURCE model consisted of three high-level steps which are explained below. First, multiple prediction models were built using Cox regression models. The models were validated in patients diagnosed in a single year and were constructed based on records from previous years. For example, records from patients diagnosed in 2012 were used to validate a prediction model based on records from patients diagnosed up to 2012 (i.e., 2005 through 2011). This was repeated for each validation cohort and therefore a total of ten prediction models were constructed. Second, the validation results for these ten models were meta-analysed to investigate the model overfit. Third, the final SOURCE prediction model was created based on the complete dataset.

### 4.1. Predictor Selection and Delphi Consensus

A set of possible predictors in the NCR dataset was established. Variables with more than 50% missing values, variables with the same value for all patients (which are therefore non-informative) and nominal variables with less than 50 cases for each category were discarded from the NCR dataset. All other variables remained as possible model predictors. 

A modified two-round Delphi consensus, similar to the COMM-PACT study in metastatic pancreatic cancer, was performed to extend this set with possibly important predictors that were missed [23]. A systematic review on prognostic factors in advanced oesophagogastric cancer served as a basis for the Delphi consensus procedure [14]. All corresponding authors of 41 phase III trials included in the systematic review were invited to participate in this study. During the first round, the experts received a list of 56 possible predictors of overall survival in metastatic oesophagogastric cancer, obtained from the systematic review [14]. For each predictor the number of studies investigating its effect and the estimated effect sizes were given. The experts were free to select as many predictors of overall survival as they deemed necessary, stratified by tumour location and treatment if needed, and were given the opportunity to include additional predictors.

After the first Delphi round, all predictors that were selected by at least 50% of the experts were included in the consensus list. Predictors selected by 20% to 50% of the experts and additional predictors that were suggested by the experts, were presented during the second consensus round alongside the results of the first round. Again, predictors selected by at least 50% of the experts in the second round were included in the final consensus list. Subsequently, all selected predictors on the consensus list were added to the set of possible predictors if available in the NCR dataset or if the predictors could be derived from other variables.

The set of possible NCR variables and predictors selected by the experts in the Delphi consensus formed the initial set of predictors. During the model specifications, predictors were selected from this joint set.

### 4.2. Development and Validation of the Prediction Model

**Figure 5:**
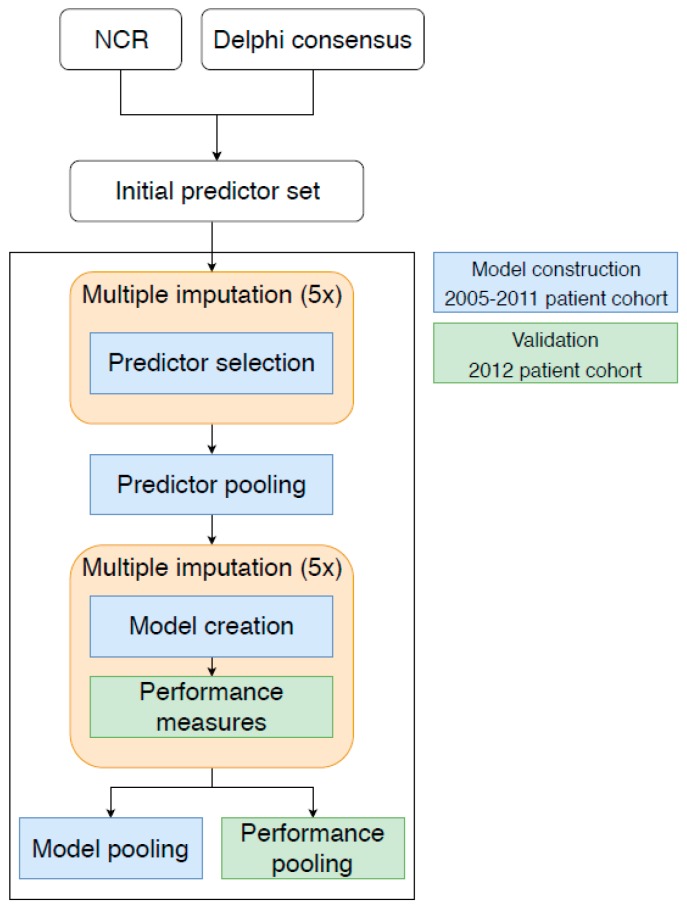
Example model creation and validation. The figure shows the construction and validation of a prediction model. This method was used during temporal cross-validation and construction of the final model. The image illustrates in this particular case the model construction based on the 2005–2011 patient cohort (shown in blue) and validated in the 2012 patient cohort (shown in green). An initial predictor set is created with variables from the NCR and extended with predictors from the Delphi consensus. We used multiple imputation for the handling of missing data after which predictors were selected by the bidirectional Akaike’s Information Criterion (AIC) procedure. Since the predictors selected by the AIC procedure may differ in each imputation, the model predictors were pooled by selecting the predictors occurring in the majority of imputations (in at least three out of five imputations). For each imputation, a model was created and validated on the 2012 patient cohort. The model parameters were pooled to establish the model for this cohort, and likewise the performance measures were pooled. This procedure was employed for all internal-external temporal validations; the model was validated on a patient cohort diagnosed in a single year and constructed on a patient cohort of all patients diagnosed in earlier years. For the final SOURCE model, the complete cohort is used for construction and validation of the model.

For the development of the prediction model, a Cox proportional hazard model with overall survival as the main outcome was developed using the regression modelling strategy (RMS) package in the R-studio environment with R version 3.3.4 [24,25,26]. An overview of the model development process is shown in Figure 5. 

To increase model generalisability and robustness, an internal-external temporal cross-validation was employed [15]. With this scheme, the data were split into so-called folds according to the patient diagnosis year. For each fold, the model was evaluated on a patient cohort diagnosed in a single year and the model was constructed on the data of all patients from earlier diagnosis years, thus mimicking a true temporal external validation. Within each fold, multiple imputation (*m* = 5) by chained equations was used to handle missing data [27]. Conditional multiple imputation was employed to transform TNM-variables from the sixth edition used for patients diagnosed prior to 2010, to the seventh edition used for patients diagnosed as of 2010 [28,29]. Specifically, these transformations were as follows: For oesophageal cancer, cN1 was transformed into cN1/cN2/cN3 and cM1A was transformed to cN1/cN2/cN3 and cM0. For gastric cancer, cN1 was transformed to cN2/cN3 and cT2 into cT2/cT3. With these transformations, the meaning of the cTNM variables remained consistent across the entire dataset, while the uncertainty of the transformations was taken into account by multiple imputation.

For each fold, bidirectional selection was performed using the AIC procedure to select from the initial predictor set including the predictors suggested during Delphi procedure [30]. Interactions between the predictor set and ‘initial treatment’ were subsequently added if the AIC statistic improved. Due to the stochastic nature of multiple imputations, the predictor selections could differ in each of the five multiple imputation rounds. Predictor pooling therefore took place by including predictors only if they were selected in at least three out of five multiple imputation rounds.

The Cox regression models were subsequently constructed for each imputation using the selected predictors. The concordance-index (c-index), calibration slope, intercept and deviance measured the model’s performance and were obtained for both the development and validation cohorts. The c-index is a measure of discrimination and ranges from 0.5 (no discrimination at all) to 1 (perfect discrimination) [31]. Calibration measures the goodness-of-fit and is described by the agreement between predicted and observed outcomes at the median overall survival time (5.1 months for oesophageal cancer and 3.9 months for gastric cancer) [32]. A linear model is used to describe this congruence and has an intercept of 0 and slope of 1 when the predictions are perfect [32]. The calibration deviance is determined by the average absolute deviance between the predicted and observed survival [33].

Finally, the performance results were pooled across all five imputations for each fold. The Cox regression models were combined into a single prediction model with pooled parameter values. The performance measures were subsequently meta-analysed with a random-effects model across all folds to obtain the internal-external validation scores. The performance measures were calculated on data in the validation cohort as well as the full model, thus an estimation of the model overfit can be made.

The construction of the full SOURCE prediction model followed identical steps. However, the complete dataset was used to construct and validate the model and the data were therefore not split into folds. 

### 4.3. Research Ethics

According to the Central Committee on Research involving Human Subjects, this type of study does not require approval from an ethics committee in the Netherlands. However, the study was approved by the Privacy Review Board of the Netherlands Cancer Registry (project code K17134).

## 5. Conclusions

In conclusion, the SOURCE prediction model for overall survival in metastatic oesophageal and gastric cancer was created based on a large nationwide cohort. SOURCE has both a fair discrimination and indicates a good accordance between predicted and observed survival. SOURCE can be used in clinical practice to give patients a personalized insight into their prognosis and thereby stimulate shared decision making.

## Figures and Tables

**Table 1:** Overview of patient characteristics stratified per tumour location. NOS: Not otherwise specified. CI: 95% confidence interval, IQR: Inter-quarter range, SD: Standard deviation. cT stage, cN stage and differentiation grade defined are according to the TNM staging system, 7th edition. *: Conditional variable imputation (see Section 4. Materials and Methods), these patients had non-missing TNM 6th variables which were transformed to the indicated TNM 7th edition stages.

Variable	Oesophagus	Gastric
**N (deaths)**	8,010 (7,825)	4,763 (4,673)
**Median overall survival in months (IQR)**	5.1 (2.2–10.1)	3.9 (1.7–8.4)
**Age (mean (sd))**	66.80 (10.91)	68.58 (12.34)
**Sex (%)**		
Male	6,284 (78.5)	2,858 (60.0)
Female	1,726 (21.5)	1,905 (40.0)
**cT stage (%)**		
Missing	1 (0.0)	1 (0.0)
cT1	108 (1.3)	58 (1.2)
cT2	1,388 (17.3)	659 (13.8)*
cT3	1,822 (22.7)	672 (14.1)*
cT4	694 (8.7)	802 (16.8)
cTX	3,997 (49.9)	2,571 (54.0)
**cN stage (%)**		
Missing	1 (0.0)	0 (0.0)
cN0	2,127 (26.6)	2,366 (49.7)
cN1	2,502 (31.2)*	1,012 (21.7)*
cN2	2,391 (29.9)*	1,264 (27.0)*
cN3	989 (12.3)*	121 (2.5)
**Primary oespohageal tumour topography (%)**		
Cervical	44 (0.5)	
Upper thoracic	205 (2.6)	
Mid-thoracic	713 (8.9)	
Lower thoracic	4,461 (55.7)	
Overlapping lesion	315 (3.9)	
Junction	2,112 (26.4)	
NOS	160 (2.0)	
**Primary gastric tumour topography (%)**		
Fundus		162 (3.4)
Corpus		954 (20.0)
Antrum Pylori		1,075 (22.6)
Pylorus		239 (5.0)
Lesser curvature NOS		181 (3.8)
Greater curvature NOS		106 (2.2)
Overlapping lesion		1,645 (34.5)
NOS		401 (8.4)

**Table 2:** List of the prediction model predictors. The variables selected by the experts are shown in the left column and variables selected for the final prediction models in the middle and right columns. Predictors indicated in bold were available in the Netherlands Cancer Registry (NCR) dataset and could be used for the creation of the SOURCE prediction model.

	Delphi Consensus	SOURCE Oesophagus Model	SOURCE Gastric Model
Age	X	X	X
Sex			X
cT stage		X	X
cN stage		X	X
Topography of primary tumour	X	X	
Histological type	X	X	
Tumour differentiation grade		X	X
Lymph node metastasis in head/neck area		X	
Intra-thoracic lymph node metastasis			X
Intra-abdominal lymph node metastasis		X	X
Only distant lymph node metastasis		X	X
Liver metastases	X	X	
Peritoneal metastases	X	X	
Number of metastatic sites	X	X	
Initial treatment	X	X	X
Peritoneal metastases with ascites	*X*		
Performance status	*X*		
Histology (lauren)	*X*		
Weight loss	*X*		
**Tumour Microsatellite Instability (MSI) status**	*X*		
Region/country	*X*		
HER status	*X*		
Disease status(unresectable vs recurrent)	*X*		
Bilirubin	*X*		

**Table 3:** Prediction model for overall survival in patients with metastatic oesophageal cancer. Initial treatment interactions terms are given in italics. NOS: Not otherwise specified. CI: 95% confidence interval. IT: Initial treatment.

Metastatic Oesophageal Cancer Prediction Model	
Covariate	Hazard Ratio (CI)
**Age**	**1.001 (0.996–1.005)**
**cT stage**	
cT1	1
cT2	1.204 (0.983–1.474)
cT3	1.103 (0.901–1.349)
cT4	1.459 (1.182–1.800)
cTX	1.459 (1.197–1.777)
**cN stage**	
cN0	1
cN1	0.974 (0.918–1.034)
cN2	1.030 (0.969–1.096)
cN3	1.154 (1.061–1.255)
**Tumour topography**	
Cervical	1
Upper thoracic	1.039 (0.744–1.450)
Mid-thoracic	0.989 (0.723–1.351)
Lower thoracic	1.062 (0.779–1.447)
Overlapping lesion	1.226 (0.886–1.697)
Junction	0.999 (0.730–1.367)
NOS	1.181 (0.837–1.665)
**Histological type**	
Adenocarcinoma	1
Squamous cell	1.011 (0.942–1.085)
Other	1.168 (1.005–1.358)
**Differentiation grade**	
G1	1
G2	0.949 (0.825–1.090)
G3	1.124 (0.981–1.288)
G4	1.396 (1.051–1.854)
**Lymph node metastasis in head/neck area**	
No	1
Yes	0.868 (0.790–0.954)
**Intra-thoracic lymph node metastasis**	
No	1
Yes	0.548 (0.430–0.698)
**Intra-abdominal lymph node metastasis**	
No	1
Yes	0.834 (0.742–0.938)
**Only distant lymph node metastasis**	
No	1
Yes	0.788 (0.732–0.849)
**Liver metastasis**	
No	1
Yes	1.222 (1.156–1.292)
**Peritoneal metastasis**	
No	1
Yes	1.274 (1.158–1.401)
**Number of metastatic sites**	1.347 (1.270–1.429)
**Initial treatment (IT)**	
None	1
Chemotherapy	0.237 (0.151–0.372)
Radiotherapy (primary tumour)	0.238 (0.151–0.375)
Radiotherapy (metastasis)	0.386 (0.169–0.884)
Chemoradiation	0.246 (0.042–1.455)
Chemotherapy + short-term radiation	0.280 (0.110–0.715)
Resection (metastasis)	0.029 (0.004–0.227)
Stent	0.881 (0.313–2.478)
Other	0.121 (0.058–0.250)
*IT = Chemotherapy*	
* Intra-thoracic lymph node metastasis*	*1.798 (1.255–2.577)*
* Intra-abdominal lymph node metastasis*	*1.091 (0.935–1.275)*
* Age*	*1.005 (0.999–1.011)*
* Number of metastatic sites*	*0.825 (0.760–0.895)*
*IT = Radiotherapy (primary tumour)*	
* Intra-thoracic lymph node metastasis*	*1.481 (1.080–2.031)*
* Intra-abdominal lymph node metastasis*	*1.266 (1.086–1.476)*
* Age*	*1.009 (1.003–1.015)*
* Number of metastatic sites*	*0.910 (0.836–0.990)*
*IT = Radiotherapy (metastasis)*	
* Intra-thoracic lymph node metastasis*	*0.972 (0.354–2.668)*
* Intra-abdominal lymph node metastasis*	*1.432 (0.963–2.130)*
* Age*	*1.009 (0.997–1.020)*
* Number of metastatic sites*	*0.901 (0.790–1.028)*
*IT = Chemoradiation*	
* Intra-thoracic lymph node metastasis*	*4.522 (0.594–34.393)*
* Intra-abdominal lymph node metastasis*	*4.407 (0.588–33.038)*
* Age*	*1.005 (0.981–1.031)*
* Number of metastatic sites*	*0.746 (0.459–1.212)*
*IT = Chemotherapy + short-term radiation*	
* Intra-thoracic lymph node metastasis*	*0.940 (0.495–1.784)*
* Intra-abdominal lymph node metastasis*	*0.921 (0.689–1.231)*
* Age*	*1.004 (0.991–1.018)*
* Number of metastatic sites*	*0.819 (0.706–0.949)*
*IT = Resection (metastasis)*	
* Intra-thoracic lymph node metastasis*	*7.155 (0.947–53.490)*
* Intra-abdominal lymph node metastasis*	*1.089 (0.385–3.084)*
* Age*	*1.038 (1.005–1.071)*
* Number of metastatic sites*	*0.810 (0.541–1.213)*
*IT = Stent*	
* Intra-thoracic lymph node metastasis*	*2.640 (1.175–5.931)*
* Intra-abdominal lymph node metastasis*	*1.027 (0.737–1.430)*
* Age*	*1.001 (0.988–1.014)*
* Number of metastatic sites*	*1.025 (0.871–1.206)*
*IT = Other*	
* Intra-thoracic lymph node metastasis*	*1.195 (0.623–2.291)*
* Intra-abdominal lymph node metastasis*	*0.889 (0.685–1.153)*
* Age*	*1.019 (1.009–1.029)*
* Number of metastatic sites*	*1.229 (1.056–1.431)*

**Table 4:** Prediction model for overall survival in patients with metastatic gastric cancer. Initial treatment interactions terms are given in italics. NOS: Not otherwise specified. CI: 95% confidence interval. IT: Initial treatment.

Metastatic Gastric Cancer Prediction Model	
Covariate	Hazard Ration (CI)
**Age**	**1.003 (0.999–1.007)**
Sex	
Male	1
Female	0.953 (0.898–1.012)
**cT stage**	
cT1	1
cT2	0.928 (0.704–1.223)
cT3	0.856 (0.650–1.128)
cT4	0.995 (0.756–1.309)
cTX	1.013 (0.775–1.324)
**cN stage**	
cN0	1
cN1	0.900 (0.834–0.971)
cN2	0.996 (0.927–1.071)
cN3	0.957 (0.793–1.156)
**Differentiation grade**	
G1	1
G2	1.294 (1.049–1.596)
G3	1.524 (1.245–1.865)
G4	1.734 (1.223–2.459)
**Intra-thoracic lymph node metastasis**	
No	1
Yes	0.739 (0.628–0.870)
**Intra-abdominal lymph node metastasis**	
No	1
Yes	0.902 (0.811–1.003)
**Only distant lymph node metastasis**	
No	1
Yes	0.771 (0.694–0.856)
**Number of metastatic sites**	1.335 (1.247–1.430)
**Initial treatment (IT)**	
None	1
Chemotherapy	0.436 (0.287–0.664)
Radiotherapy (primary tumour)	1.428 (0.363–5.619)
Radiotherapy (metastasis)	8.419 (1.754–40.411)
Chemotherapy + short-term radiation	1.268 (0.138–11.611)
Resection (primary tumour)	0.427 (0.169–1.080)
Resection (metastasis)	0.092 (0.027–0.313)
Stent	1.441 (0.132–15.795)
Other	0.422 (0.143–1.250)
*IT = Chemotherapy*	
* Age*	*1.000 (0.994–1.006)*
* Number of metastatic sites*	*0.864 (0.786–0.949)*
*IT = Radiotherapy (primary tumour)*	
* Age*	*0.990 (0.974–1.007)*
* Number of metastatic sites*	*0.918 (0.681–1.239)*
*IT = Radiotherapy (metastasis)*	
* Age*	*0.976 (0.958–0.995)*
* Number of metastatic sites*	*0.706 (0.516–0.965)*
*IT = Chemotherapy + short-term radiation*	
* Age*	*0.990 (0.962–1.020)*
* Number of metastatic sites*	*0.717 (0.507–1.015)*
*IT = Resection (primary)*	
* Age*	*0.999 (0.987–1.011)*
* Number of metastatic sites*	*0.955 (0.717–1.271)*
*IT = Resection (metastasis)*	
* Age*	*1.025 (1.009–1.042)*
* Number of metastatic sites*	*0.879 (0.668–1.156)*
*IT = Stent*	
* Age*	*0.997 (0.968–1.027)*
* Number of metastatic sites*	*0.957 (0.656–1.396)*
*IT = Other*	
* Age*	*1.012 (0.998–1.027)*
* Number of metastatic sites*	*0.803 (0.625–1.032)*

**Table 5:** Performance measures for the SOURCE in oesophagus and gastric cancer. The discrimination index and calibration statistics are shown side-by-side for both the complete SOURCE model as well as for the internal-external temporal validation. The 95% confidence interval is stated in parentheses for each outcome.

	Oesophageal Cancer		Gastric Cancer	
	Complete Model	Internal-External Validation	Complete Model	Internal-External Validation
**c-index**	0.713 (0.705–0.720)	0.706 (0.698–0.714)	0.686 (0.677–0.696)	0.676 (0.665–0.686)
**calibration slope**	1.006 (1.005–1.007)	1.017 (0.962–1.071)	0.987 (0.985–0.989)	1.009 (0.891–1.127)
**calibration intercept**	−0.002 (−0.003–0.002)	−0.020 (−0.053–0.013)	−0.006 (−0.006–-0.005)	−0.011 (−0.058–0.036)
**calibration deviance**	0.002 (0.002–0.002)	0.021 (0.011–0.035)	0.011 (0.011–0.011)	0.031 (0.021–0.042)

## Supplementary Materials

The following are available online at http://www.mdpi.com/2072-6694/11/2/187/s1. Table S1: Overview of included and excluded variables during the modified Delphi. The left column indicates in green the variables included in round one by a majority of participants, and in red the variables which were excluded. The centre column shows variables which were included by 20%–50% of participants during the first round as well as newly proposed variables. The right column indicates for the second round again the included and excluded variables based on the variables in the centre column. Table S2: Overview of additional patient characteristics stratified per tumour location. CI: 95% confidence interval. Initial treatment: Defined as the treatment with the earliest starting date after initial diagnosis of metastasized oesophagogastric cancer; treatments that were started within three days thereafter were jointly regarded as ‘initial treatment’. This period of three days was extended to five days in case of chemoradiation and 28 days to include chemotherapy + short-term radiation.
