# Supplementary material for: SOURCE: A Registry-Based Prediction Model for Overall Survival in Patients with Metastatic Oesophageal or Gastric Cancer
Source: Cancers (Basel). 2019 Feb 5;11(2):187. doi: 10.3390/cancers11020187 (PMC6406639; doi:10.3390/cancers11020187)
Supplement: Supplementary file 1 [file cancers-11-00187-s001.pdf]

Supplementary Material: SOURCE: A Registry-Based Prediction Model for Overall Survival in Patients with Metastatic Oesophageal or Gastric Cancer

Héctor G. van den Boorn, Ameen Abu-Hanna, Emil ter Veer, Jessy Joy van Kleef, Florian Lordick, Michael Stahl, Jaffer A. Ajani, Rosine Guimbaud, Se Hoon Park, Susan J. Dutton, Yung-Jue Bang, Narikazu Boku, Nadia Haj Mohammad, Mirjam A.G. Sprangers, Rob H.A. Verhoeven, Aeilko H. Zwinderman, Martijn G.H. van Oijen, and Hanneke W.M. van Laarhoven

Table 1. Overview of included and excluded variables during the modified Delphi. The column "Round 1 Included" and "Round 1 Rejected" indicate in green the variables included in round one by a majority of participants, and in red the variables which were excluded. The column "Round 1 Possible Predictors" and "Newly Proposed Predictors" show variables which were included by 20%–50% of participants during the first round as well as newly proposed variables. The column "Round 2 Included" and "Round 2 Rejected" indicate for the second round again the included and excluded variables based on the variables in the "Round 1 Possible Predictors" and "Newly Proposed Predictors" column.

ROUND 1 INCLUDED	% selected
Peritoneal metastasis	100%
Surgery of primary tumour	100%
Esophagus vs stomach tumour	100%
Performance status	88%
Liver metastasis	88%
Disease status (locally advanced vs metastasis)	88%
Histology (lauren)	75%
Number of metastatic sites	75%
Region/country	63%
HER status	63%
Age	50%
Disease status (unresectable vs recurrent)	50%
Histology (adenocarcinoma vs squamous cell carcinoma)	50%
Bilirubin	50%
**ROUND 1 REJECTED**	-
White blood cell count	13%
Quality of life (physical functioning)	13%
Quality of life (role functioning)	13%
Macroscopic tumour type (borrmann)	13%
Aspartate aminotransferase (AST)	13%
Tumor size	13%
Opt	13%
Vascular endothelial growth factor a (VEGFA)	13%
Neuropilin	13%
MET expression	13%
Pulmonary metastasis	0%

Tuberous sclerosis	0%
Sodium	0%
Pylorus intact	0%
ERCC expression	0%
Lymphnode metastasis	0%
Peritoneal metastasis without drip infusion	0%
Dihydropyrimidine dehydrogenase (DPD)	0%
Thymidine phosphorylase (TP)	0%
Epidermal growth factor receptor (EGFR)	0%
Lymphocytes	0%
Neutrophils	0%
Body surface area (BSA)	0%
Sparc expression	0%
Clinical N stage	0%
**ROUND 1 POSSIBLE PREDICTORS**	% selected
Alkaline phosphatase level	38%
GEJ vs stomach tumour	38%
Gender	38%
Neutrophil to lymphocyte ratio	38%
Peritoneal metastasis with ascites	38%
Measurable disease	25%
Quality of life (global health status)	25%
Lactate dehydrogenase (LDH) levels	25%
Creactive protein (crp)	25%
Ethnicity	25%
Prior chemotherapy	25%
Prior radiation	25%
Weight loss	25%
Charlson comorbidity index	25%
High vegf low ang	25%
Viceral (lung or liver) metastasis	25%
Kras wild type (vs mutation)	25%
**NEWLY PROPOSED PREDICTORS**	-
Bone metastasis	-
Oral intake	-
Brain metastasis	-
Tumor microsatellite instability (MSI) status	-
Body mass index (BMI)	-
Pain scale	-
EBV tumor status	-
Cirrhosis	-
**ROUND TWO INCLUDED**	% selected
Peritoneal metastasis with ascites	63%

Weight loss	50%
Tumor microsatellite instability (MSI) status	50%
**ROUND 2 REJECTED**	
GEJ vs stomach tumour	38%
Neutrophil to lymphocyte ratio	38%
Bone metastasis	38%
Brain metastasis	38%
EBV tumor status	38%
Alkaline phosphatase level	25%
Gender	25%
Creactive protein (crp)	25%
Prior radiation	25%
Measurable disease	13%
Quality of life (global health status)	13%
Lactate dehydrogenase (LDH) levels	13%
Prior chemotherapy	13%
Kras wild type (vs mutation)	13%
Oral intake	13%
Body mass index (BMI)	13%
Ethnicity	0%
Charlson comorbidity index	0%
High vegf low ang	0%
Viceral (lung or liver) metastasis	0%
Pain scale	0%
Cirrhosis	0%

Table 2. Overview of additional patient characteristics stratified per tumour location. CI: 95% confidence interval. Initial treatment: Defined as the treatment with the earliest starting date after initial diagnosis of metastasized oesophagogastric cancer; treatments that were started within three days thereafter were jointly regarded as 'initial treatment'. This period of three days was extended to five days in case of chemoradiation and 28 days to include chemotherapy + short-term radiation.

Variable	Oesophagus	Gastric
**N (deaths)**	8,010 (7,825)	4,763 (4,673)
**Histological type (%)**		
Adenocarcinoma	6,321 (78.9)	4,691 (98.5)
Squamous cell	1,423 (17.8)	0 (0.0)
Other	266 (3.3)	72 (1.5)
**Tumour differentiation grade (%)**		
Missing	3,472 (43.3)	2,180 (45.8)
G1	112 (1.4)	42 (0.9)
G2	1,464 (18.3)	488 (10.2)
G3	2,896 (36.2)	2,028 (42.6)
G4	66 (0.8)	25 (0.5)
**Lymph node metastasis in head/neck area**		
Missing	267 (3.3)	168 (3.5)
No	7232 (90.3)	4538 (95.3)
Yes	511 (6.4)	57 (1.2)
**Intra-thoracic lymph node metastasis**		
Missing	267 (3.3)	168 (3.5)

No	7487 (93.5)	4419 (92.8)
Yes	256 (3.2)	176 (3.7)
**Intra-abdominal lymph node metastasis**		
Missing	267 (3.3)	168 (3.5)
No	6218 (77.6)	3973 (83.4)
Yes	1525 (19.0)	622 (13.1)
**Only distant lymph node metastasis (%)**		
Missing	267 (3.3)	168 (3.5)
No	6,532 (81.5)	4,141 (86.9)
Yes	1,211 (15.1)	454 (9.5)
**Liver metastasis (%)**		
Missing	267 (3.3)	168 (3.5)
No	3,699 (46.2)	2,873 (60.3)
Yes	4,044 (50.5)	1,722 (36.2)
**Peritoneal metastasis (%)**		
Missing	267 (3.3)	168 (3.5)
No	7,190 (89.8)	2,735 (57.4)
Yes	553 (6.9)	1,860 (39.1)
**Number of metastatic sites (%)**		
Missing	267 (3.3)	168 (3.5)
1	4,457 (55.6)	3,099 (65.1)
2	2,208 (27.6)	1,067 (22.4)
3 or more	1,078 (13.5)	429 (9.0)
**Initial treatment (%)**		
None	2,131 (26.6)	2,266 (47.6)
Chemotherapy	2,216 (27.7)	1,648 (34.6)
Radiotherapy (primary tumour)	2,081 (26.0)	154 (3.2)
Radiotherapy (metastasis)	367 (4.6)	63 (1.3)
Chemoradiation	80 (1.0)	0 (0.0)
Chemotherapy + short-term radiation	317 (4.0)	52 (1.1)
Resection (primary tumour)	0 (0.0)	247 (5.2)
Resection (metastasis)	56 (0.7)	97 (2.0)
Stent	298 (3.7)	56 (1.2)
Other	464 (5.8)	180 (3.8)
